# Outcomes of Laparoscopic Surgery of Deep Infiltrating Endometriosis: A Prospective Cohort Study in Yas Infertility Center, Tehran -

**DOI:** 10.31661/gmj.v14i.3899

**Published:** 2025-12-27

**Authors:** Fatemeh Davari Tanha, Mahbod Ebrahimi, Fatemeh Salehi, Nooshin Faraji, Shaghayegh Norozi Larki

**Affiliations:** ^1^ Department of Infertility, Yas Hospital, Tehran University of Medical Sciences, Tehran, Iran

**Keywords:** Endometriosis, Irritable Bowel Syndrome, Quality of Life, IVF

## Abstract

**Background:**

Endometriosis is a prevalent medical condition that significantly affect the quality of life in a substantial proportion of women, affecting their fertility and manifesting with many symptoms like gastrointestinal symptoms. The present study aimed to evaluate the impact of laparoscopic surgery for endometriosis on general and sexual quality of life, and gastrointestinal symptoms in patients diagnosed with deep infiltrating endometriosis (DIE).

**Materials and Methods:**

This prospective cohort study was conducted on 129 women diagnosed with DIE-associated infertility and referred to Yas Infertility Center in Tehran , from 2022 to 2023. Demographic data and the presence and severity of gastrointestinal symptoms were recorded. These symptoms were reassessed one month following endometriosis surgery. Participants were also asked to complete the SF-36 Quality of Life Questionnaire and Female Sexual Function Index (FSFI) questionnaires before the surgical procedure and one month later. Data were analyzed using SPSS version 24.

**Results:**

This study of 129 patients (mean age 34.82±5.83 years, BMI 25.64±3.77 kg/m²) revealed near-universal ovarian involvement (99.2%) and adhesions (99.2%), primarily affecting rectosigmoid (24.1%) and cervix (29.5%). Pre-intervention, 98.4% reported abdominal pain (47.3% severe), 37.9% bloating, and 24.8% constipation. Post-intervention showed dramatic improvements: pain-free cases rose to 90.7%, bloating reduced to 9.3%, and constipation to 6.2% (all P0.0001). The FSFI score increased (57.6±28.8 to 65.2±27.4, P0.0001), and SF-36 pain scores halved (6.8±1.2 to 2.7±0.9, P0.0001), though vitality and emotional well-being remained unchanged. Surgical complications included hemoglobin drop (17.8%) and intestinal injury (6.2%).

**Conclusion:**

The findings of this study indicate that laparoscopic treatment of DIE not only alleviates gastrointestinal symptoms but also significantly enhances the quality of life and sexual function in affected women.

## Introduction

Endometriosis is a chronic, recurrent, and disabling condition affecting approximately 5% to 10% of women [1]. Endometriotic lesions are characterized by ectopic endometrial glands and stroma outside the uterine cavity, commonly involving the ovaries and other pelvic structures [2]. These lesions trigger a persistent inflammatory response, forming scar tissue and adhesions [3]. Depending on the location of the lesions, endometriosis may impair the function of the bowel or bladder [4]. The clinical presentation of endometriosis varies widely. While some women may remain asymptomatic, most experience dysmenorrhea, dyspareunia, and chronic fatigue [5]. A considerable proportion of patients also report gastrointestinal symptoms. These may include severe abdominal pain, constipation, bloating, flatulence, psychological distress affecting daily life, urgency in defecation, and a sensation of incomplete bowel evacuation [6].

Endometriosis and gastrointestinal manifestations are two prevalent medical conditions that significantly affect the quality of life in a substantial number of women, adolescent girls, and even some postmenopausal women [6]. Affected individuals commonly experience menstrual irregularities, infertility, abdominal and pelvic pain, and disrupted bowel movements. Moreover, approximately 61% of women and adolescent girls are diagnosed with irritable bowel syndrome (IBS) [6]. Given the chronic inflammatory nature of both conditions, endometriosis and IBS share considerable symptom overlap. A recent nationwide study in the United States demonstrated that endometriosis increases the risk of developing IBS by nearly threefold. However, it remains unclear whether endometriosis independently contributes to bowel involvement or serves as a distinct risk factor for IBS [4]. Several theories have been proposed to explain the association between endometriosis and IBS. These include immunological mechanisms involving mast cell activation, abnormal levels of inflammatory cytokines, and heightened activity of immune cells within the peritoneal cavity, observed in both conditions. Fagervold et al. reported a direct correlation between the severity of gastrointestinal symptoms and the depth of endometriotic infiltration into the bowel; symptoms improved following surgical excision of the lesions [7].

Endometriosis is a chronic condition, and like other chronic diseases, early diagnosis plays a critical role in its management by improving prognosis and enhancing patients’ quality of life. The gold standard for diagnosing endometriosis involves surgical methods; however, due to the invasive nature and high cost of laparoscopy, such approaches are typically reserved for specific clinical situations. As a result, pelvic examination and imaging techniques are more commonly employed to diagnose suspected cases of endometriosis [8]. On the other hand, there is no universally accepted standard for diagnosing gastrointestinal symptoms. The most commonly used diagnostic frameworks are the Manning and Rome criteria. Consequently, definitively identifying gastrointestinal symptoms can vary depending on the diagnostic criteria. Patients with endometriosis report a wide range of gastrointestinal symptoms, which often overlap significantly with those observed in irritable bowel syndrome (IBS), making the differential diagnosis particularly challenging.

In the study conducted by Malin et al., patients with endometriosis reported significantly worsened abdominal pain, constipation, bloating, and urgency in defecation [4]. Similarly, Khadija Saidi et al. reported that women diagnosed with endometriosis were two to three times more likely to experience gastrointestinal symptoms [9]. According to findings by Nicolas Bourdel et al., endometriosis adversely affects physical, psychological, and social health, with a negative impact on health-related quality of life in these patients [8]. Ballester et al. demonstrated that colorectal surgery for endometriosis followed by ICSI-IVF represents a favorable treatment strategy for women with established infertility, even in cases where prior ICSI-IVF attempts had failed [10]. Given the importance of this issue, the present study was designed to evaluate the impact of laparoscopic surgery for endometriosis on general and sexual quality of life, gastrointestinal symptoms, and IVF success in patients diagnosed with DIE .

## Materials and Methods

The present cross-sectional study (Ethics Code: IR.TUMS.MEDICINE.REC.1402.017) was conducted after obtaining approval from the Ethics Committee of Tehran University of Medical Sciences. A total of 129 women diagnosed with endometriosis who were referred to Yas Infertility Center were enrolled. Patients with diagnosis of DIE-associated infertility were included if diagnosis was confirmed by medical and imaging findings. Exclusion criteria included a history of usage of immunosuppressive medications, presence of malignancy, adenomyosis of the uterus, or severe underlying medical conditions that contraindicated surgery, fertility, or IVF treatment. Following history taking and clinical examinations, demographic data were recorded using a researcher-designed questionnaire before surgery. Based on the number of eligible patients referred to Yas Hospital and using the following sample size formula, considering a reported prevalence of gastrointestinal symptoms ranging from 70% to 77% in patients with endometriosis, the required sample size was calculated to be 129 participants. A convenience sampling method was employed in this study.


n = \frac{Z_{1-\alpha/2}^{2} p q}{d^{2}}

### Data Collection Instruments

Initially, demographic information, endometriosis-related parameters, including anatomical involvement and associated findings, were documented through surgical and imaging reports. Colonoscopy results were obtained from endoscopic evaluations. Medical histories were verified through patient interviews and chart reviews. The severity and presence of gastrointestinal symptoms were recorded. These symptoms were reassessed following the surgical procedure. In addition, participants were asked to complete the SF-36 and FSFI questionnaires before and one month after surgery. The SF-36 Quality of Life Questionnaire consists of 36 items covering eight domains: physical functioning (10 items), role limitations due to physical health problems (4 items), role limitations due to emotional problems (3 items), vitality (4 items), emotional well-being (5 items), social functioning (2 items), pain (2 items), and general health perceptions (5 items). Items are measured using various Likert scales, including dichotomous, 3-point, 5-point, and 6-point formats. The total score ranges from 0 to 100, with higher scores indicating better quality of life. This questionnaire was initially developed by Ware and Sherbourne in 1992 in the United States, and its validity and reliability have been confirmed across different patient populations [11]. The reliability and validity of the Persian version were also assessed by Dr. Montazeri and colleagues, reporting a Cronbach’s alpha of 0.79 [12].

The Female Sexual Function Index (FSFI) is a validated instrument widely used to assess female sexual function. It consists of 19 items scored on a 6-point Likert scale and evaluates six domains: sexual desire (2 items), arousal (4 items), lubrication (4 items), orgasm (3 items), satisfaction (3 items), and pain (3 items). Higher scores indicate better sexual functioning. Each domain has a maximum score of 6; the total scale score can reach 36. A score of zero indicates no sexual activity during the past four weeks. The FSFI was developed by Rosen in 2004, and its validity and reliability have been confirmed [13]. Dr. Mohammadi and colleagues assessed the Persian version's reliability and validity, reporting a Cronbach’s alpha of 0.83 [14].

### Study Procedure and Follow Ups

All eligible patients diagnosed with endometriosis referred to Yas Hospital from 2022 to 2023 (1401-1402 in the Iranian calendar) were enrolled in this study. Inclusion criteria consisted of women diagnosed with endometriosis and DIE with a surgical indication, as well as those presenting with gastrointestinal symptoms. The presence or absence of gastrointestinal symptoms, including abdominal pain, bloating, defecation disorders, constipation, diarrhea, cramping, nausea, and vomiting, was also documented in the same questionnaire before the surgical intervention.

Gastrointestinal symptoms were reassessed one month postoperatively to determine improvement or persistence of symptoms, and their association with IVF outcomes was evaluated. Additionally, changes in general and sexual quality of life before and after surgery were assessed using the SF-36 and FSFI questionnaires.

Six months later, documents were assessed and patents were called to ask about succuss of IVF, defined by pregnancy successfully being conceived and confirmed by chemical analysis.

### Data Analysis

All data were entered into SPSS software , version 24. Descriptive statistics for qualitative variables were presented using frequency tables and charts, while quantitative data were summarized using mean and standard deviation. For inferential analysis, the Chi-square test was used to examine associations between qualitative variables, and the independent t-test was employed to compare quantitative variables. A P-value of less than 0.05 was considered statistically significant.

## Results

**Table:** Table1. Endometriosis-related Findings in Patients

**Variable**	**Mean ± SD or n (%)**	**Variable**	**Mean ± SD or n (%)**
**Mean age (years)**	34.82 ± 5.83	**Gravidity**	
**Mean BMI (kg/m²)**	25.637 ± 3.77	**Nulligravida**	61 (52.7%)
**Intramural involvement in DIE**	7 (5.4%)	**≥1 pregnancy**	68 (47.3%)
**Ovarian involvement**	128 (99.2%)	**Medical History**	
**Presence of adhesions in DIE**	128 (99.2%)	**No history**	79 (61.2%)
**Presence of kissing ovaries**	76 (58.9%)	**Hypothyroidism**	16 (12.4%)
**Müllerian anomalies**	3 (2.3%)	**Hypertension**	5 (3.9%)
**Sites of DIE involvement**		**Diabetes**	2 (1.6%)
**- Rectum**	27 (20.9%)	Other*	27 (20.9%)
**- Uterosacral ligament**	3 (2.3%)	**Colonoscopy Findings**	
**- Left ovary**	16 (12.4%)	**Normal**	99 (76.7%)
**- Right ovary**	15 (11.6%)	**Abnormal**	30 (23.3%)
**- Cervix and posterior cervix**	38 (29.5%)	**Specific Colonoscopy Findings**	
**- Posterior cul-de-sac**	5 (3.9%)	**Erythema**	16 (12.4%)
**- Rectosigmoid**	31 (24.1%)	**Stricture**	22 (17.1%)
**- Posterior vaginal wall**	9 (6.9%)	**Mucosal thickening**	7 (5.4%)
**- Rectovaginal septum**	8 (6.2%)	**Mucosal bleeding**	7 (5.4%)

**Table:** Table2. Changes in Clinical Gastrointestinal Symptoms Before and After Intervention

**Symptom**	**Before Intervention**	**After Intervention**	**P-value**
	**Positive n (%)**	**Positive n (%)**	
**Rectal bleeding**	13 (10.1%)	0 (0.0%)	-
**Constipation**	32 (24.8%)	8 (6.2%)	< 0.0001
**Abdominal bloating**	49 (37.9%)	12 (9.3%)	< 0.0001
**Diarrhea**	5 (3.9%)	0 (0.0%)	-
**Dyspepsia**	36 (27.9%)	26 (20.2%)	< 0.0001
**Reflux**	27 (20.9%)	14 (10.9%)	< 0.0001
**Abdominal Pain**			
**No pain**	2 (1.6%)	117 (90.7%)	< 0.0001
**Mild (Grade 1-2)**	8 (6.2%)	10 (7.7%)	
**Moderate (Grade 3)**	58 (44.9%)	1 (0.8%)	
**Severe (Grade 4-5)**	61 (47.3%)	1 (0.8%)	

A total of 129 eligible patients were included in the study, with a mean age of 34.82 ± 5.83 years and a mean body mass index (BMI) of 25.64 ± 3.77 kg/m². Data on patients’ findings associated with endometriosis are presented in Table-1 . Comorbidities like hypothyroidism (12.4%) and hypertension (3.9%) were reported, with 61.2% having no medical history. DIE related findings of near-universal ovarian involvement (99.2%) and adhesions (99.2%) were prevalent. Common DIE sites included the rectosigmoid (24.1%) and cervix/posterior cervix (29.5%), while müllerian anomalies were rare (2.3%). Colonoscopy revealed 76.7% normal findings, though strictures (17.1%) and erythema (12.4%) were notable. Frequency of gastrointestinal symptoms were as follows: rectal bleeding (10.1%), constipation (24.8%), abdominal pain (98.4%), abdominal bloating (37.9%), diarrhea (3.9%), dyspepsia (27.9%), and reflux (20.9%) before the intervention.

A total of 16 patients (12.4%) experienced surgical complications , that the highest complication rate was due to hemoglobin drop that happened in 17.8% of patients while only 1.5% needed blood transfusion. intestinal injury happened in 6.2%.

Changes in patients’ clinical findings before and after the intervention are presented in Table-2. Rectal bleeding, initially reported in 10.1% of cases, was completely eliminated post-treatment. Similarly, diarrhea, which affected 3.9% of participants at baseline, disappeared entirely after the intervention. Constipation saw a dramatic drop from 24.8% to just 6.2%, a change that was highly statistically significant (P<0.0001). Likewise, abdominal bloating improved substantially, falling from 37.9% to 9.3% (P<0.0001). While dyspepsia and reflux also decreased (27.9% to 20.2% and 20.9% to 10.9%, respectively), their improvements, though significant (P<0.0001), were less pronounced than other symptoms. The most striking transformation was seen in abdominal pain. Before treatment, nearly all patients (98.4%) experienced some level of pain, with 47.3% suffering severe (Grade 4-5) discomfort. After the intervention, 90.7% were pain-free, and severe cases plummeted to just 0.8% (P<0.0001). Even moderate pain (Grade 3), initially affecting 44.9%, was reduced to a mere 0.8%.

The results regarding changes in FSFI scores, the overall score, and individual domains of the SF-36 questionnaire before and after the intervention are presented in Table-3. The intervention led to remarkable improvements in both sexual function and overall quality of life. The FSFI total score, reflecting female sexual health, increased significantly from 57.6 ± 28.8 to 65.2 ± 27.4 (P<0.0001), suggesting enhanced sexual well-being after treatment. For SF-36 domains, the most dramatic change was seen in pain, which dropped by more than half (from 6.8 ± 1.2 to 2.7 ± 0.9, P<0.0001). Physical functioning also improved notably, rising from 10.2 ± 0.5 to 11.5 ± 1.3 (P<0.0001), while general health perceptions saw a meaningful boost (12.0 ± 1.3 to 13.9 ± 1.9, P<0.0001). Interestingly, role limitations due to physical health decreased (6.7 ± 1.7 to 4.2 ± 0.7, P<0.0001), possibly indicating fewer activity restrictions post-intervention. A similar but smaller reduction occurred in emotional role limitations (4.2 ± 1.4 to 3.4 ± 1.0, P<0.0001). However, some areas remained unchanged: Vitality (energy/fatigue), emotional well-being, and social functioning showed no statistically significant differences (P>0.05), suggesting these aspects were less affected by the intervention. Despite this, the total SF-36 score still improved significantly (65.9 ± 3.3 to 68.0 ± 3.5, P<0.0001) Overall, 67.44% of individuals attempting pregnancy successfully conceived.

## Discussion

**Table:** Table3. Changes in FSFI Scores and SF-36 Total and Domain Scores Before and After Intervention

**Questionnaire**	**Score Type**	**Before Intervention Mean ± SD **	**After Intervention ± SD **	**P-value**
**FSFI**	Total Score	57.628 ± 28.760	65.217 ± 27.445	< 0.0001
	Physical Functioning	10.178 ± 0.458	11.504 ± 1.306	< 0.0001
	Role Limitations Due to Physical Health	6.744 ± 1.719	4.233 ± 0.724	< 0.0001
	Role Limitations Due to Emotional Problems	4.178 ± 1.449	3.395 ± 1.019	< 0.0001
**SF-36**	Vitality (Energy/Fatigue)	12.349 ± 1.412	12.124 ± 1.867	0.137
	Emotional Well-being	13.868 ± 1.486	13.899 ± 1.802	0.872
	Social Functioning	5.566 ± 0.759	5.543 ± 0.729	0.796
	Pain	6.814 ± 1.217	2.729 ± 0.864	< 0.0001
	General Health	11.985 ± 1.299	13.884 ± 1.873	< 0.0001
	Total SF-36 Score	65.946 ± 3.313	67.989 ± 3.468	< 0.0001

In this study, analysis of the SF-36 questionnaire domains revealed a significant improvement in physical functioning and general health scores following surgical intervention. Conversely, scores related to role limitations due to physical health, role limitations due to emotional problems, and pain significantly decreased after surgery. These findings are broadly consistent with those reported by Ballester and Meuleman [15]. The study by Viganò et al. highlighted that irritable bowel syndrome (IBS) and endometriosis are two conditions that independently or concurrently affect a substantial proportion of the female population and have significant implications for their quality of life. Their association may not be purely epidemiological but could reflect a shared pathophysiological basis, which is supported by overlapping mechanisms such as mast cell activation, neuroinflammation, dysbiosis, and increased intestinal permeability—all of which are exacerbated in individuals with endometriosis. Recognizing this association is crucial due to the high prevalence of both conditions and their shared clinical presentation, particularly chronic abdominal pain [16].

Lea et al. reported that both irritable bowel syndrome (IBS) and endometriosis are common conditions; however, gastrointestinal manifestations of endometriosis are relatively rare. In many patients initially diagnosed with IBS, subsequent laparoscopy revealed the presence of endometriosis and even small bowel obstruction. These findings highlight the importance of considering alternative diagnoses, particularly endometriosis, in patients with an initial IBS diagnosis when there is diagnostic uncertainty [17]. In another study, Lee et al. identified several factors associated with the severity of gastrointestinal symptoms in patients with endometriosis. These included early-stage endometriosis, mood disorders, tenderness during physical examination, a history of sexual abuse, and sleep disturbances [18]. In the present study, several distressing gastrointestinal symptoms—including constipation, abdominal pain, bloating, dyspepsia, and reflux—showed significant improvement following surgical treatment for endometriosis. Similarly, Schomacker et al. demonstrated that women with endometriosis experience more frequent and more severe gastrointestinal symptoms compared to women without the condition. Their cross-sectional study, which collected online questionnaire responses from 373 women, found that the prevalence of gastrointestinal symptoms was significantly higher in women with endometriosis (OR=5.32). Bowel involvement was also more common in this group. The study concluded that proper treatment and management of endometriosis can substantially reduce the severity of gastrointestinal symptoms and, in some cases, lead to complete resolution [19]. The findings of both studies, as mentioned above, are in complete agreement with the present research results, further supporting the effectiveness of endometriosis treatment in alleviating or eliminating gastrointestinal symptoms.

In a study by Nnoaham et al. conducted on 1,418 women aged 18 to 45 years with endometriosis, it was reported that endometriosis adversely affects HRQoL and work productivity across countries and ethnicities. Nevertheless, women face diagnostic delays in primary care settings [20]. The present study's findings align with this research, showing that surgical intervention for endometriosis significantly improved general and sexual health scores, with notable improvements in physical functioning, emotional well-being, and pain reduction. Similarly, Fourquet et al. reported that endometriosis-related symptoms, such as chronic disabling pelvic pain and infertility, negatively and substantially affect physical and psychological health, health-related quality of life, and work productivity in women. Their findings also emphasized that proper management and treatment of endometriosis can significantly improve physical and sexual quality of life [21]. These results are consistent with those of the current study and demonstrate that effective management of endometriosis can substantially enhance quality of life, particularly in physical, emotional, and psychological domains.

Moore et al. also reported that women with endometriosis are frequently misdiagnosed with irritable bowel syndrome (IBS) before receiving an accurate diagnosis. Among 160 women who met the Rome III criteria for gastrointestinal symptoms, 36% were found to have concurrent endometriosis [22]. The severity and stage of endometriosis may influence the success rates of assisted reproductive technologies (ART), particularly in vitro fertilization (IVF) [23]. Various studies have reported inconsistent results regarding IVF success rates in patients with endometriosis. Some studies have found no significant difference in pregnancy outcomes between women with and without endometriosis undergoing IVF [18]. However, a survey by Hamdan et al. in 2015 demonstrated that the treatment of endometriosis could significantly affect IVF success rates. The live birth rate among women with untreated endometriosis undergoing IVF was 27.7%, compared to 43.6% in those undergoing early-stage treatment and 46.3% in those at the final stages of endometriosis management [24].

In a prospective cohort study by Bianchi et al., conducted on 179 infertile women diagnosed with both endometriosis and irritable bowel syndrome, IVF outcomes were compared in women with DIE-associated infertility who underwent extensive laparoscopic resection of endometriosis before IVF. The study demonstrated that comprehensive laparoscopic excision of DIE significantly improved IVF pregnancy rates in women with DIE. Moreover, it was observed that surgical treatment of DIE also led to a considerable reduction in the severity of gastrointestinal symptoms [25].

## Conclusion

Surgical treatment of endometriosis, particularly DIE, significantly reduces the severity of various gastrointestinal symptoms such as constipation, abdominal pain, bloating, dyspepsia, and reflux. It also leads to a meaningful improvement in quality of life, especially in the domains of physical functioning and general health, as well as in the sexual function of women undergoing assisted reproductive treatments like IVF. However, due to the considerable overlap in clinical manifestations between endometriosis and irritable bowel syndrome, a high level of clinical vigilance is required when evaluating patients presenting with such symptoms. Both conditions should be carefully considered in the differential diagnosis. Larger-scale and more comprehensive studies are recommended to enhance diagnostic accuracy and improve differentiation between these two disorders.

## Conflict of Interest

None.
